# Phase II study of adjuvant chemotherapy with S1 plus oxaliplatin for Chinese patients with gastric cancer

**DOI:** 10.1186/s12885-018-4480-9

**Published:** 2018-05-09

**Authors:** Guoxiu Wang, Jiuda Zhao, Yan Song, Wen Zhang, Yongkun Sun, Aiping Zhou, Jing Huang, Feng Du, Lin Yang

**Affiliations:** 1 Department of Medical Oncology, National Cancer Center/Cancer Hospital, Chinese Academy of Medical Sciences and Peking Union Medical College, Beijing, 100021 China; 2 Department of Medical Oncology, Yangquan No.1 People’s Hospital, Shanxi, 045000 China; 3 Affiliated Hospital of Qinghai University, Affiliated Cancer Hospital of Qinghai University, Xining, 810000 China; 4 The VIPII Gastrointestinal Cancer Division of Medical Department, Peking University Cancer Hospital and Institute, Beijing, 100142 China

**Keywords:** Gastric cancer, S-1, Oxaliplatin, Adjuvant chemotherapy

## Abstract

**Background:**

S-1 plus oxaliplatin(SOX) has been demonstrated to be effective and well tolerated for patients with metastatic gastric cancer. We conducted this phase II study to evaluate the feasibility of SOX as adjuvant chemotherapy for gastric cancer after curative resection.

**Methods:**

Adjuvant chemotherapy consisted of six to eight cycles of S-1 plus oxaliplatin. Oxaliplatin was administered intravenously at a dose of 130 mg/m^2^ on day 1. S-1 was administered orally at a dose of 70 mg/m^2^ daily from day 1 to 14 of a 3-week cycle. A total of 58 patients were enrolled in this study. The primary end point of the trial was the treatment completion rate for six cycles. Secondary endpoints were safety, 1-year and 3-year of disease free survival (DFS) and overall survival (OS).

**Results:**

A total of 58 patients were enrolled and 54 patients have been analysed. The completion rate of six cycles was 72.2%. Grade 4 toxicities included neutropenia (1.9%) and thrombocytopenia (3.7%). Grade 3 toxicities included leukopenia (5.6%), neutropenia (24.1%), thrombocytopenia (13.0%), nausea (7.4%), vomiting 13.0%), and diarrhea (13.0%). There was no grade 3 or higher peripheral sensory neuropathy and treatment-related death. The median follow-up time was 42.4 months. 1-year and 3-year DFS rate were 85.2 and 75.9%, respectively.1-year and 3-year OS were 98.1 and 85.2%, respectively.

**Conclusion:**

Adjuvant chemotherapy for GC with S-1 plus oxaliplatin is safe and feasible in Chinese patients. The optimal dose of oxaliplatin and optimal cycles of treatment still need to be further investigated.

**Trial registration:**

clinicaltrials.gov identifier NCT01542294. Trial registration date: 03/02/2012.

## Background

Gastric cancer (GC) is the fourth most common malignancy and the second leading cause of cancer related death in the world [1]. Curative treatments for GC remain a challenge with 988,000 new cases and 73,000 deaths each year [2]. In China, GC is the second-leading cause of cancer death for both men and women [3]. Surgical resection is the only curative treatment option for localized disease. However, locally advanced (stage II–III) GC often recurs, even after curative resection is performed. Therefore, adjuvant systemic chemotherapy plays a crucial role in improving the prognosis in patient with resectable GC.

S-1 is an oral anticancer agent containing tegafur (FT), 5-chloro-2,4-dihydroxipyridine (CDHP) and potassium oxonate (Oxo) at a molar ratio of 1:0.4:1. It showed approximately 40% of response rate in phase II trials in patients with advanced or recurrent GC [4, 5]. Based on the results from the ACTS-GC trial [6, 7], S-1 monotherapy for 1 year was established as the standard postoperative adjuvant chemotherapy for GC in Japan. However, a subgroup analysis of data demonstrated that both disease free survival (DFS) and overall survival (OS) of patients with stage IIIb and IV was not improved. It is urgent to investigate new treatment strategy.

For recurrent or metastatic GC, combined with S-1 and cisplatin (SP) showed improved progression free survival (PFS) and OS compared with S-1 alone [8]. But in adjuvant setting, SP was demonstrated too toxic as a post-gastrectomy treatment [9, 10]. For patients with GC, oxaliplatin was more tolerable than cisplatin, without compromising the antitumor effect, which was evidenced by the data from one randomized clinical trial demonstrating a favourable safety profile and equivalent benefit in patients who received S-1 plus oxaliplatin versus those with S-1 and cisplatin [11].

In China, SOX regimen with 130 mg/m^2^ oxaliplatin was commonly used. Our data showed that the SOX regimen with 130 mg/m^2^ oxaliplatin was a promising regimen as the first-line chemotherapy in patients with advanced GC [12]. As adjuvant chemotherapy for postoperative GC, when combined with 130 mg/m^2^ oxaliplatin every 3 weeks, 70 mg/m^2^/day S-1 for 2 weeks was the optimal dose in our phase I study [13, 14]. We conducted this phase II study to further evaluate the tolerability and safety of SOX as adjuvant therapy after curative resection in Chinese patients with GC.

## Methods

### Study design and patients

This is a single-arm, prospective, phase 2 study conducted at Cancer Institute and Hospital of Chinese Academy of Medical Sciences (Beijing, China) in accordance with the Helsinki Declaration and the good clinical practice guidelines. The study protocol was approved by the ethics committee of Cancer Hospital, Chinese Academy of Medical Sciences and Peking Union Medical College (approval number 11–41/476). All patients provided written informed consent before starting of the study. This study was registered on clinicaltrials.gov on March 2, 2012 (Identifier: NCT01542294).

The inclusion criteria were as follows: histologically confirmed stage II-III gastric adenocarcinoma (according to the 7th edition of the American Joint Committee on Cancer tumor-node-metastasis system); R0 surgery with D2 or more extensive lymphadenectomy; can start chemotherapy within 6 weeks after surgery; no prior chemotherapy or radiotherapy; sufficient oral intake; age distribution of 20 to70 years old; an Eastern Cooperative Oncology Group (ECOG) performance status of 0–1; adequate bone marrow, renal and liver function, including an absolute granulocyte counts of > 1500/L; a platelet counts of > 100,000/L; a hemoglobin level of > 90 g/L; a serum bilirubin level of less than the upper limit of normal (ULN); a normal creatinine level; an alanine transaminase and aspartate transaminase level of < 1.5 × ULN; electrocardiogram was normal. Patients with ascites or evidence of peritoneal, hepatic or distant metastases were excluded from this study. Patients were also excluded if they had mental abnormalities, severe comorbid conditions, severe drug hypersensitivity or peripheral sensory neuropathy.

### Treatment and assessment

Chemotherapy consisted of six to eight cycles of S-1 plus oxaliplatin. All treatment cycles were administered every 3 weeks. S-1 was administered orally twice daily at a dose of 70 mg/m^2^/day from day 1 to 14. Oxaliplatin was infused intravenously for 2 h on day 1 at a dose of 130 mg/m^2^. Before infusion of oxaliplatin, antiemetics (e.g., a 5-hydroxytryptamine3 receptor antagonist and dexamethasone) were administered prophylactically to prevent nausea and vomiting. If patients developed grade 4 haematological toxicities, or grade 3–4 diarrhea, laryngeal mucositis, peripheral sensory neuropathy and palmer-planter erythrodysestheia syndrome, the doses of both oxaliplatin and S-1 were reduced by 20%. The dose of oxaliplatin was reduced if the platelet count was less than 75,000/mm^3^ on day 23 with delayed initiation of the next treatment cycle or if grade 2 peripheral sensory neuropathy was noted on the first day of the next cycle. S-1 and oxaliplatin could be reduced twice, but treatment was discontinued if subsequent reduction was indicated. In the cases of oxaliplatin-related peripheral sensory neuropathy, S-1 could be continued as monotherapy. But oxaliplatin monotherapy was not allowed if S-1 was discontinued.

During the study, complete blood count and blood chemistry studies were performed before initiation of each cycle. Computed tomography or magnetic resonance image were performed as baseline before chemotherapy. Then, the presence or absence of disease recurrence was evaluated every 3 months for 2 years, then every 6 months for 3 years. Adverse events were evaluated using the National Cancer Institute-Common Toxicity Criteria version 4.0.

### Statistical analysis

The primary end point was the treatment completion rate for six cycles of SOX therapy. Treatment completion was defined as the percentage of patients who completed six cycles of SOX therapy and included patients who continued with S-1 monotherapy after discontinuation of oxaliplatin treatment. The point estimate and 95% confidence interval (CI) were calculated. Secondary endpoints were safety, 1-year and 3-year of DFS and OS.

The required sample size was calculated according to our previous work and a phase 3 trial. Our phase 2 trial of SOX as a fist-line chemotherapy for patients with advanced GC shown that the median number of cycles for the SOX regimen was five (range, 2–8). In addition, the actual treatment completion rates of adjuvant treatmen as capecitabine plus oxaliplatin for GC in the CLASSIC study was 67%. Therefore, the SOX regimen was considered as feasible if the point estimate of the treatment completion rate was not lower than 70% in this study. Considering the expected treatment completion rate was 70.0% and the range of the accurate 95% CI of the point estimate for the treatment completion rate was 26% or less, the required sample size was estimated to be 53 patients. Thus, the target enrollment was 58 patients, in order to make accommodations for 10% dropout rate.

The treatment completion rate was defined as the percentage of patients who completed all the six cycles of SOX therapy, and included patients who continued with S-1 monotherapy after discontinuation of oxaliplatin therapy. A safety analysis was conducted in patients who received at least one dose of S-1 or oxaliplatin. The treatment completion rate and the ratio of delivered dose to the planned dosage administration were analyzed for the patients who met the inclusion criteria. Patient characteristics, feasibility, adverse events, DFS and OS were analysed. DFS was defined as the time from the date of surgery to the date of documented recurrence or death from any cause, or last follow-up, whichever came first. OS was defined as the time from the date of surgery to the date of death from any cause, or last follow-up. Survival curves were estimated using the Kaplan-Meier method. All analyses were carried out using an SPSS 23.0 software package (SPSS Inc., Chicago, IL, USA).

## Results

### Patient characteristics

Between May 2011 and April 2014, 58 patients were enrolled to the study. Among them, 3 did not receive allocated intervention, 1 did not meet inclusion criteria. 54 patients were included in the safety analysis and the full analysis set. The patient characteristics are summarized in Table 1. There were 37 males and 17 females. The median age was 55 years (range, 30–70 years). The body surface area (BSA) ranged from 1.35 to 2.06 m^2^. All patients exhibited a good performance status (ECOG 0–1). Total gastrectomy and partial gastrectomy were performed in 8(14.8%) and 47 (85.2%) patients, respectively.11 (20.4%) had stage IIA disease, 11 (20.4%) had stage IIB disease, 12 (22.2%) had stage IIIA, 12 (22.2%) had stage IIIB, 8(14.8%) had stage IIIC disease.

**Table 1:** Patient characteristics *n* = 54

Characteristic					
Age(years)			Type of gastrectomy		
	median	55		total	8
	range	30–71		partial	45
Gender				combined resection	2
	male	37	Tumor position		
	female	17		cardia	8
PS(ECOG)				fundus ventriculi	10
	0	14		corpus ventriculi	25
	1	40		autrum pyloricum	11
BSA(m^2^)			T stage		
	median	1.7		pT1	4
	range	1.35–2.06		pT2	6
Pathological type				pT3	24
	intestinal	14		pT4	20
	diffuse	15	N stage		
	mixed	17		pN0	10
	unknow	8		pN1	14
Differentiation				pN2	16
	moderately	7		pN3a	11
	low	45		pN3b	3
	unknown	2	TNM stage		
Vessel tumor emboli				Ia	1
	no	29		Ib	2
	yes	24		IIa	8
	unknown	1		IIb	11
Nerve invasion				IIIa	12
	no	28		IIIb	12
	yes	25		IIIc	7
	unknown	1		IV^a^	1

*BSA* body surface area, *ECOG PS* Eastern Cooperative Oncology Group performance status

^a^peritoneal metastasis(P1)

### Compliance

Thirty-nine patients completed six cycles of treatment, and the treatment completion rate was 72.2% (95% CI 60.25–84.15%), and 26 patients (48.1%) completed eight cycles of treatment (Tables 2 and 3), which was consistent with our prior study [11]. The treatment completion rate was 67.57% for male and 82.35% for female, 77.78% for patients who underwent total gastrectomy and 71.11% % for those who underwent partial gastrectomy, 74.07% for < 55 years-old patients, 69.57% for 55–65 years-old patients and 75.00% for 65 years or older.

**Table 2:** Chemotherapy completion rate *n* = 54

No. of cysles	Complete chemotherapy		Complete SOX	
	No. (%)	95%Cl	No. (%)	95%Cl
1	54(100)	1.00,1.00	54(100)	1.00,1.00
2	51(94.4)	0.88,1.01	51(94.4)	0.88,1.01
3	47(87.0)	0.78,0.96	45(83.3)	0.73,0.93
4	45(83.3)	0.73,0.93	40(74.1)	0.62,0.86
5	44(81.5)	0.71,0.92	37(68.5)	0.56,0.81
6	39(72.2)	0.60,0.84	31(57.4)	0.44,0.71
7	27(50)	0.37,0.63	13(24.1)	0.13,0.36
8	26(48.1)	0.35,0.61	12(22.2)	0.11,0.33

**Table 3:** Tolerability of subgroups

	n	Treatment completion rate (%)		Relative dose intensities(%)			
		6 cycle (%)	8 cycle (%)	mean		median	
				S-1 (%)	Oxaliplatin (%)	S-1 (%)	Oxaliplatin (%)
Age(years)							
< 55	27	20(74.07)	15(55.56)	68.74(98.20)	124.11(95.47)	68.00(97.14)	127.00(97.69)
55~ 65	23	16(69.57)	9(39.13)	69.60(99.43)	123.74(95.18)	70.00 (100.00)	125.00 (96.15)
≥65	4	3 (75.00)	2 (50.00)	69.75 (99.64)	116.25 (89.42)	70.00 (100.00)	116.50 (89.62)
Gender							
Male	37	25 (67.57)	17 (45.95)	69.38 (99.11)	122.32 (94.10)	70.00 (100.00)	124.00 (95.38)
Female	17	14 (82.35)	9 (52.94)	68.77 (98.24)	125.65 (96.65)	68.00(97.14)	129.00 (99.23)
PS(ECOG)							
0	14	10 (71.43)	6 (42.86)	68.64 (98.06)	125.29 (96.37)	68.50 (97.86)	127.50 (98.08)
1	40	29 (72.50)	20 (50.00)	69.38 (99.11)	122.70 (94.38)	70.00 (100.00)	123.00 (94.62)
BSA							
< 1.7	26	22 (84.62)	13 (50.00)	70.85 (101.21)	123.96 (95.36)	71.00 (101.43)	125.00 (96.15)
≥1.7	28	17 (60.71)	13 (46.43)	67.64 (96.63)	122.82 (94.48)	68.00(97.14)	126.00 (96.92)
Type of gastrectomy							
Total	9	7 (77.78)	4 (44.44)	70.33 (100.48)	121.22 (93.25)	71.00 (101.43)	119.00 (91.54)
Partial	45	32 (71.11)	22 (48.89)	68.96 (98.51)	123.80 (95.23)	68.00(97.14)	126.00 (96.92)

Fifteen patients (27.8%) could not complete the six cycles of treatment and the reason for discontinuation were as follows: seven patients discontinued therapy because of adverse events (AEs) (thrombocytopenia in 5 patients, vomiting in 2 patients,); six patients withdrew informed consent;t wo patients were detected retroperitoneal lymph node metastasis.

Nine patients (16.7%) experienced one level of S-1 dose reduction, and no patient required two level of S-1 dose reduction. Twenty-one patients (38.9%) experienced one level of oxaliplatin dose reduction, and 1 patient (1.9%) required two level of dose reduction. Forty-seven (87%) patients experienced treatment delay of initiation subsequent cycle. The main reason of dose reduction and treatment-delay were AEs including thrombocytopenia, neutropenia vomiting and peripheral sensory neuropathy.

The most common reasons for discontinuation of oxaliplatin by investigators decision were thrombocytopenia and peripheral sensory neuropathy. The median relative dose intensities were 98.6% for S-1 and 96.5% for oxaliplatin.

### Safety

All of the 54 patients were assessable for AEs (Table 4). Grade 3 AEs occurred in 36 (66.7%) patients and grade 4 AEs occurred in 3(5.6%) patients. There were no treatment-related deaths. The most common hematological toxicities of all grades include leucopenia (75.9%) and neutropenia (72.2%) and thrombocytopenia (61.1%). Grade 3/4 hematological toxicities were leucopenia (5.6%), neutropenia (26%) and thrombocytopenia (16.7%). Grade 4 neutropenia and thrombocytopenia occurred in 1(1.9%) and 2(3.7%) patients, respectively. Among non-hematological toxicity of all grades, nausea was the most frequent (77.8%) followed by peripheral sensory neuropathy (61.1%), asthenia (53.7%) and vomiting (38.9%). Grade 3 non-hematological toxicity included vomiting (13.0%), nausea (7.4%), diarrhea (1.9%). However, there were no grade 4 non-hematological adverse reactions.

**Table 4:** Drug-related adverse events *n* = 54

Adverse events					
	Grade 1 (%)	Grade 2 (%)	Grade 3 (%)	Grade 4 (%)	All grades (%)
Leukopenia	16 (29.6)	22 (40.7)	3 (5.6)	0	41 (75.9)
Neutropenia	11 (20.4)	14 (25.9)	13 (24.1)	1 (1.9)	39 (72.2)
Anemia	17 (31.5)	8 (14.8)	0	0	25 (46.3)
Thrombocytopenia	13 (24.1)	11 (20.4)	7 (13.0)	2 (3.7)	33 (61.1)
Febrile neutropenia	–	–	1 (1.9)	0	1 (1.9)
Skin hyperpigmentation	25 (46.3)	0	–	–	25 (46.3)
Malaise	29 (53.7)	0	–	–	29 (53.7)
Nausea	25 (46.3)	13 (24.1)	4 (7.4)	–	42 (77.8)
Vomiting	7 (13.0)	7 (13.0)	7 (13.0)	0	21 (38.9)
Laryngeal mucositis	2 (3.7)	0	0	0	2 (3.7)
Diarrhea	2 (3.7)	1 (1.9)	1 (1.9)	0	4 (7.4)
Peripheral sensory neuropathy	32 (59.3)	1 (1.9)	0	0	33 (61.1)
Palmer-planter erythrodysestheia syndrome	1 (1.9)	0	0	–	1 (1.9)
Alanine aminotransferase increased	9 (16.7)	3 (5.6)	0	0	12 (22.2)

### DFS and OS

**Fig. 1:**
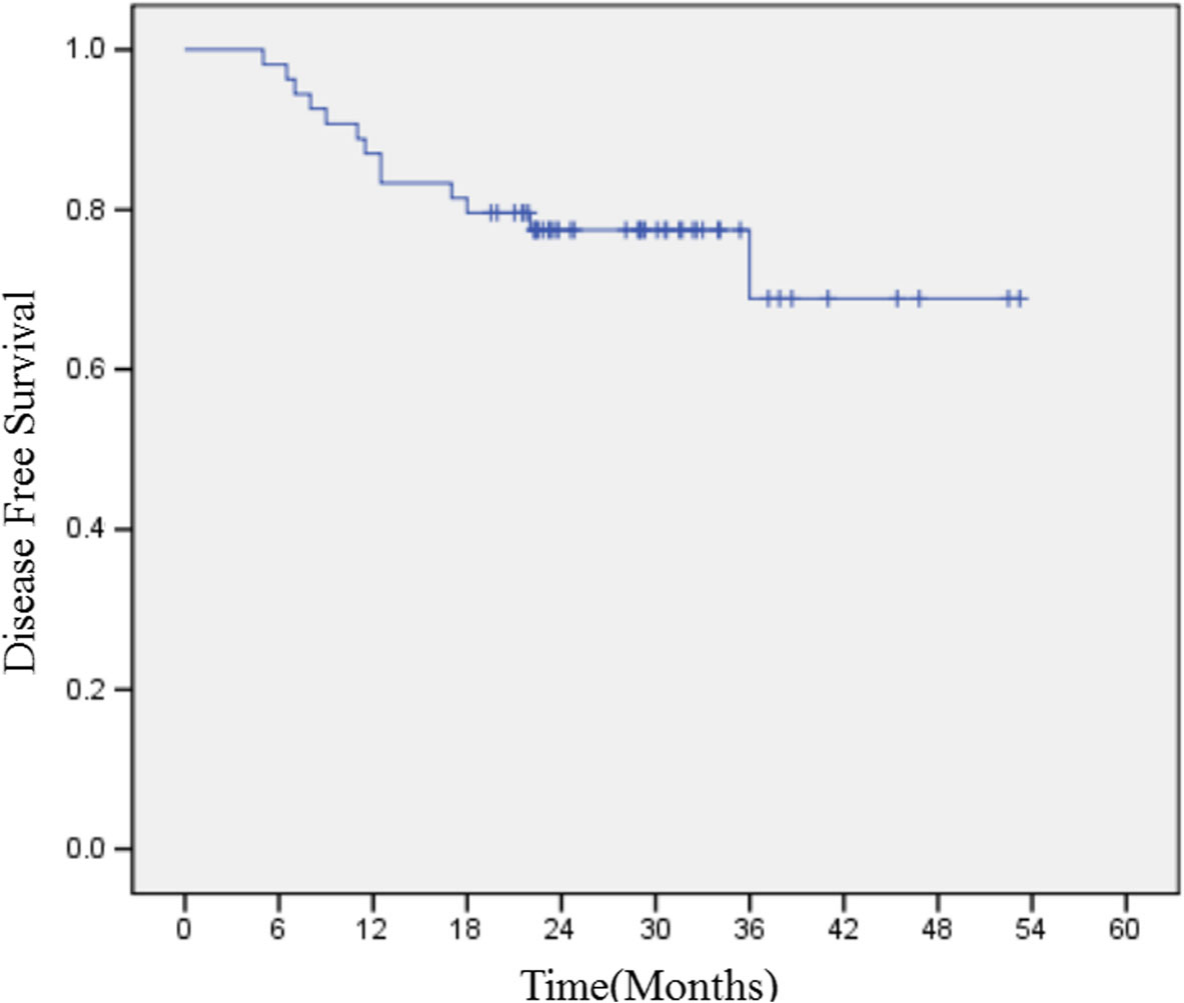
Disease-free survival curve for patients treated with adjuvant S1 plus oxaliplatin chemotherapy

**Fig. 2:**
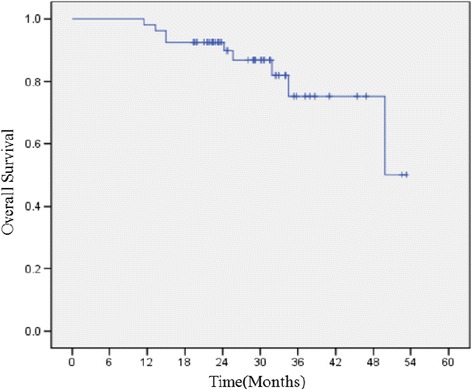
Overall survival curve for patients treated with adjuvant S1 plus oxaliplatin chemotherapy

The median follow-up time was 42.4 months (range, 37.2–47.6 months). There were 13 patients recurred. The 3-year DFS were 75.9%. The 1-year DFS were 85.2%. There were 8 patients died. The the 3-year OS were 85.2%, and 1-year OS were 98.1%. (Figs. 1 and 2).

## Discussion

To our knowledge this is the first report on the toxicity and safety analysis of oxaliplatin plus S-1 (SOX) treatment for Chinese patients with stage II/III gastric cancer who had received curative D2 gastrectomy. In the present study, the six cycles chemotherapy completion rate was 72.2% (95% CI 60.25–84.15%), similar with our previous work and other regimens reported in different trials [6, 13, 15]. The safety profile of SOX observed in the present study were comparable as previously reported in patients who received SOX regimen for advanced gastric cancer [11, 16]. The most frequent (incidence rate 10% or more) grade 3/4 AEs were neutropenia (26%), thrombocytopenia (16.7%) and vomiting (13.0%).

The main reason for discontinuation of chemotherapy is thrombocytopenia which we think mainly due to oxaliplatin. Yamada Y et al. [11] recently reported a phase III randomized trial conducted in Japan comparing SOX with SP showed that SOX is as effective as SP for AGC with favorable safety profile. The dose of oxaliplatin they used is 100 mg/m^2^ every 3 weeks. Grade 3/4 thrombocytopenia is only 10.1%, the same as SP (10.3%). So, reduction of the dosage of oxaliplatin is one way of reducing risk of grade 3/4 thrombocytopenia. Shitara K et al. [17] conducted a phase II trial to investigate the tolerability and safety of 8 cycles of SOX (oxaliplatin 100 mg/m^2^) as adjuvant chemotherapy for stage III gastric cancer. No grade 4 or higher events occurred. Grade 3 thrombocytopenia in only 4.8%. The completion rate of treatment was 74.2%. 41.9 and 61.7% patients required dose reduction of S-1 and oxaliplatin, respectively, and 82.3% patients required chemotherapy administration to be delayed mainly because of AEs. A study has conveyed that S-1 monotherapy as the first cycle, followed by 3 cycles of S-1 plus cisplatin for gastric cancer adjuvant chemotherapy is feasible and tolerable [10]. Moreover, considering chemotherapy was administrated soon after operation, the patients in this study also treated as S-1 in the first cycle, and SOX regimen after the first cycle. The authors think that such a design may contribute to the favorable treatment completion rate. However, the efficacy of such treatment was not estimated in this study.

In our study, 16.7 and 38.9% patients required dose reduction of S-1 and oxaliplatin, respectively, mainly because of AEs including thrombocytopenia, neutropenia vomiting and peripheral sensory neuropathy. The dose reduction rate is lower than the Japanese study [17]. The reason may be that the dose of S-1 70 mg/m2 we recommend is more appropriate for Chinese patients when combined with oxaliplatin 130 mg/m2. 87% patients required chemotherapy administration to be delayed, which is similar to the Japanese study [17]. In the ARTIST trial [18] comparing postoperative treatment with capecitabine plus cisplatin (XP) versus XP plus radiotherapy with capecitabine (XP/XRT/XP), treatment was completed as planned by 75.4% of patients (172 of 228) in the XP arm and the estimated 3-year DFS rates were 74.2% in the XP arm. The 3 year DFS and OS was 72.2 and 80.1% [6] for S-1 monotherapy for 1 year and 74 and 83% [15] for capecitabine plus oxaliplatin (XELOX) treatment for 6 months, respectively. In our study, 72.2% (95%CI 60–84%) of patients received at least six cycles of treatment and the 3-year DFS were 75.9% that is close to other studies. [15, 17, 19].

Although there occurred dose reduction, treatment delay and treatment discontinuation, there was not significant difference in the treatment completion rate and relative dose intensities for patients in gender, age, performance status, body surface acre and type of gastrectomy.

## Conclusions

In conclusion, adjuvant chemotherapy for GC with S-1 plus Oxaliplatin is safe and feasible in Chinese patients. The optimal dose of Oxaliplatin and optimal cycles of treatment still need to be further investigated.

## Abbreviations

AEs: Adverse events
BSA: Body surface area
CI: Confidence interval
DFS: Disease free survival
ECOG: Eastern Cooperative Oncology Group
GC: Gastric cancer
OS: Overall survival
DFS: Disease free survival
SOX: S-1 plus oxaliplatin
SP: S-1 and cisplatin
ULN: Upper limit of normal
XELOX: Capecitabine plus oxaliplatin
XP: Capecitabine plus cisplatin
